# The efficacy of a novel CO_2_ topical vapocoolant spray for reducing needle-related pain in dogs

**DOI:** 10.3389/fvets.2026.1754998

**Published:** 2026-03-10

**Authors:** Na-rae Lee, Hyun-Jung Han

**Affiliations:** 1 Department of Veterinary Emergency and Critical Care Medicine, College of Veterinary Medicine, Konkuk University, Seoul, Republic of Korea; 2 KU Center for Animal Blood Medical Science, Konkuk University, Seoul, Republic of Korea

**Keywords:** cryoanesthesia, cryotherapy, local anesthetic, needle-induced pain, vapocoolant spray, visual analog scale

## Abstract

**Introduction:**

Effective pain management is essential in veterinary needle-related procedures. Traditional methods, including infiltrative, cream, and spray formulations, have limitations such as delayed onset, inconsistent temperature control, and skin damage. This study evaluated the efficacy of a novel vapocoolant spray (VetEase^®^, Recensemedical, Hwaseong-si, Republic of Korea) for enhanced pain relief.

**Methods:**

Ninety cases of dogs received cryoanesthesia immediately before undergoing three types of needle-related procedures: centesis (including cystocentesis, thoracentesis, and abdominocentesis), fine-needle aspiration (FNA), and jugular venipuncture. Each procedure was divided into three groups based on cryoanesthesia spray conditions: control (no spray), group A (2 s at 2°C), and group B (5 s at 2°C). Modified pain scores and visual analog scale evaluations were recorded immediately after needle insertion to assess the reduction in pain. Pain evaluation criteria included vocalization, general movements, and other observable responses.

**Results:**

Significant pain reduction was observed in the centesis procedure for the group treated with cryoanesthesia at 2°C for 5 s (Group B), with a mean modified pain score of 0.8 compared to 3.7 in the control group (*p* = 0.001). The visual analog scale also showed a significant reduction in Group B (*p* = 0.001). Although both cryoanesthesia groups showed reduced pain levels during FNA and jugular venipuncture procedures, the differences did not reach statistical significance. No skin complications were reported.

**Conclusion:**

The novel cryoanesthetic device significantly alleviated needle-related pain during centesis procedures in dogs. It provided rapid local anesthesia, eliminating the need for prolonged onset times and minimizing skin complications, thereby presenting a viable alternative to topical anesthetics.

## 1 Introduction

Needle-related procedures are routinely performed in dogs for the diagnosis and treatment of various diseases in veterinary practice. These procedures include centesis (cystocentesis, thoracentesis, and abdominocentesis), fine-needle aspiration (FNA), and jugular venipuncture. These procedures can elicit pain responses and cause anxiety in veterinary patients, complicating the administration of treatments and therapeutic interventions. Therefore, effective pain management strategies are becoming increasingly important in clinical veterinary practice to minimize stress and improve patient compliance.

Several methods are traditionally used for local anesthesia, which can be broadly categorized into infiltrative, cream, and spray formulations. Infiltrative anesthesia includes the use of agents such as lidocaine and bupivacaine, cream formulations include lidocaine cream and the eutectic mixture of lidocaine and prilocaine, and spray formulations include vapocoolant sprays and 10% lidocaine spray, which provide local analgesic effects (1). The analgesic efficacy of topical anesthetic approaches may vary depending on the formulation and clinical context. Infiltrative anesthesia is generally effective in alleviating procedural pain; however, it requires needle injection, which can itself cause additional discomfort to patients. Cream formulations, particularly eutectic lidocaine–prilocaine cream, have been reported to provide superior analgesic efficacy in some studies (2, 3). However, direct comparisons between eutectic lidocaine–prilocaine cream and vapocoolant sprays in dogs have not demonstrated clear superiority of EMLA over vapocoolant-based approaches (4). In addition, the prolonged application time, typically ranging from 20 to 60 min, limits its practicality for routine clinical use (5). Therefore, there remains a clinical need for a rapid, non-invasive, and effective method for pain mitigation in veterinary practice.

Cryotherapy lowers skin temperature and subsequently slows the nerve conduction velocity of both C-fibers and A-delta fibers. These physiological effects reduce tissue nociceptor activity, thereby inducing an analgesic effect (5–7). Building on these effects, various cryoanesthetic techniques have been developed for pain management. Vapocoolant is one such method, utilizing volatile liquid refrigerants (e.g., ethylene chloride) to lower skin temperature through rapid evaporation, thereby providing cutaneous anesthesia (8). First described by Travell in 1955 for reducing injection-related pain (9), vapocoolant spray has subsequently been investigated in specific clinical contexts. In human medicine, vapocoolant spray has been shown to effectively reduce pain and anxiety associated with vaccinations, botulinum toxin injections, and intramuscular injection procedures (5, 10). In veterinary medicine, vapocoolant spray has been reported to provide analgesia by reducing the temperature of ear tissue during ear notching in calves, and it has been found to be safe and effective in reducing pain responses during joint puncture in horses (11, 12). However, previous studies have reported that the indirect application of vapocoolant spray using a swab to reduce pain during intravenous catheterization in dogs was ineffective (13). Factors such as the distance between the cooling agent and the skin and the duration of application have not been standardized in either human or veterinary medicine, and this lack of consistency in application methods may contribute to variability in the pain-reducing effects of vapocoolant sprays (14). Notably, there remains a lack of research on standardized application protocols for vapocoolant spray, particularly in companion animals.

In this context, the novel device evaluated in the present study utilizes pressurized CO_2_ gas rather than liquid refrigerants. The human-use version of this device has previously been employed to reduce pain during intralesional steroid injections and laser tattoo removal procedures (15, 16). Unlike conventional liquid-based vapocoolant sprays, the rapid expansion of CO_2_ upon release induces a strong but transient cooling effect at the skin surface, while the gas does not remain on the skin, allowing rapid dissipation of cooling. In addition, the device is designed to standardize spraying distance, angle, target temperature, and application duration, thereby minimizing operator-related variability and enabling more predictable and reproducible skin cooling.

Based on these characteristics, this study aimed to evaluate the analgesic efficacy of topical anesthesia using a novel vapocoolant spray in dogs undergoing needle-related procedures, and to determine the optimal application conditions for pain reduction. We hypothesized that application of the novel vapocoolant spray under standardized conditions would result in reduced pain responses compared with control conditions during these procedures.

## 2 Materials and methods

### 2.1 Patients and study design

A single-blind, randomized controlled trial involving 90 cases in 56 dogs was conducted at the Konkuk Veterinary Medical Teaching Hospital and the KU I'M DOgNOR Blood Donation Center (Konkuk University, Seoul, Republic of Korea) from January 2024 to September 2024.

The cases in this study comprised patients undergoing three types of needle-related procedures: (1) centesis, including cystocentesis, thoracentesis, and abdominocentesis, (2) FNA, and (3) jugular venipuncture. For vapocoolant spray application, the device used in this study is equipped with an integrated temperature sensor that continuously monitors the skin surface temperature during application. Once the target skin surface temperature of 2 °C is reached, the device maintains the skin surface temperature at this level for the preset application duration. Based on the duration for which the target temperature was maintained, procedures were categorized into two treatment groups: Group A (sprayed for 2 s at 2 °C) and Group B (sprayed for 5 s at 2 °C). A control group was included in which no vapocoolant spray was applied. Some dogs were exposed to multiple spraying conditions. For each indication, dogs were assigned to the control, 2-s, or 5-s spray conditions using a computer-generated random sequence at the time of the procedure. In dogs undergoing multiple procedures or exposed to more than one spray condition, the order of spray conditions was also randomized using the same method to minimize potential order and carryover effects.

Descriptive patient information, including age, body weight, and sex, was recorded for all dogs included in the study. To minimize potential confounding factors, patients with a score of 6/24 or higher on the Short Form Glasgow Composite Pain Scale were excluded, as those scoring above 6 were considered to require analgesic treatment for underlying conditions (17, 18). This measure was implemented to avoid confounding procedural pain with pre-existing pain. Patients who were classified as depressed, obtunded, stuporous, or comatose based on mentation scoring were excluded from the study. Furthermore, patients exhibiting hypersensitivity to the device sound, as well as those who had previously received sedatives or analgesics, were excluded from the study.

Ethical approval was granted by the Institutional Animal Care and Use Committee at Konkuk University, under approval number [KU23264].

### 2.2 Cryoanesthesia protocol for pain management during needle-related procedures

**Figure 1:**
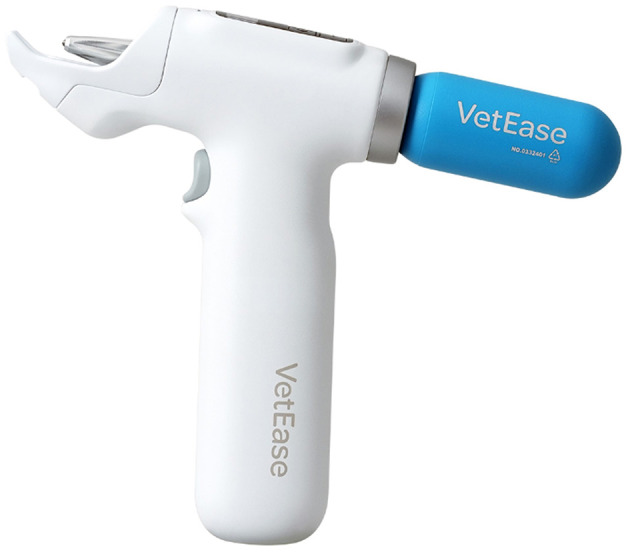
A novel vapocoolant spray for cryoanesthesia (VetEase^®^ Recensemedical, Hwaseong-si, Republic of Korea). A portable device used for cryoanesthesia. Reproduced with permission.

**Figure 2:**
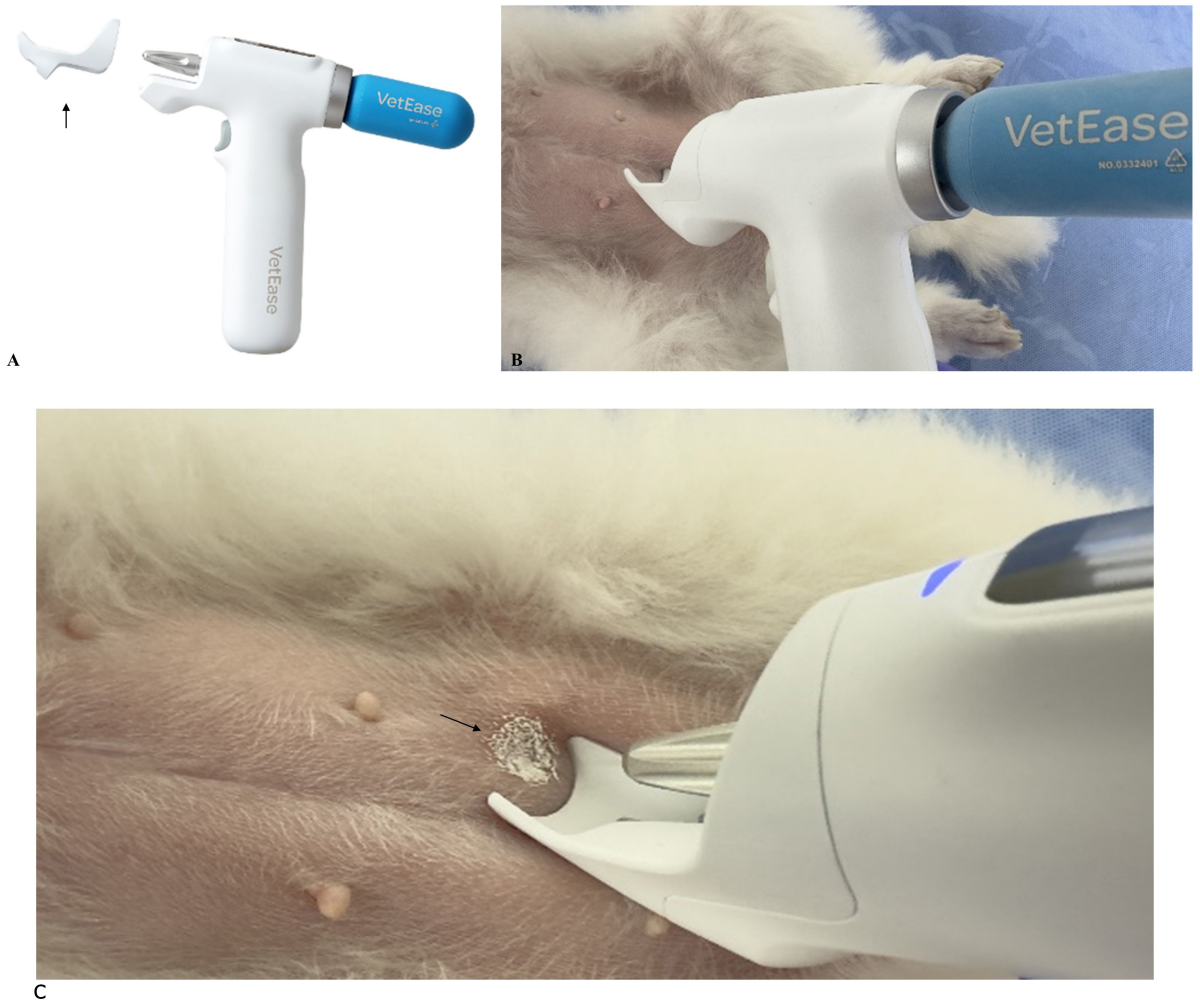
Configuration and function of the novel vapocoolant spray. **(A)** The device was fitted with a specialized guard (arrow) to ensure a 1.5 cm distance from the skin, following the manufacturer's guidelines. **(B)** Side view of the device maintaining the specified distance with the guard attached, allowing the sensor to detect skin temperature upon contact. **(C)** Cooling with a CO_2_ cartridge created a white circular area (arrow). Reproduced with permission.

For all procedures, the insertion areas were shaved and sterilized with 70% isopropyl alcohol. For cryoanesthesia, this study utilized the vapocoolant spray (VetEase^®^) (Figure 1). The device was fitted with a specialized guard to maintain a distance of 1.5 cm from the skin, as per the manufacturer's guidelines. The cooling effect, induced by a CO_2_ cartridge, resulted in a visible white circular area on the skin (Figure 2), and the treatment was performed by inserting the needle immediately after cooling or within 5 s.

In group A, the insertion sites were sprayed for 2 s at 2 °C, while group B received a 5 s spray at the same temperature (Supplementary Video 1). The control group did not receive the spray application. To prevent contamination, the skin contact tip of the device was disinfected with 70% isopropyl alcohol before and after each use.

Needle-related procedures included centesis (cystocentesis, thoracentesis, abdominocentesis), FNA, and jugular venipuncture. Centesis procedures were performed under ultrasound guidance using a 22-gauge needle. FNA was conducted using a 22-gauge needle, with samples collected from multiple directions. For jugular venipuncture, a 16-gauge catheter was inserted into the jugular vein for blood collection.

All dogs were evaluated for adverse skin reactions, including pain, bruising, erythema, pallor, pruritus, swelling, discoloration, and other potential effects at the end of the procedure and 24 h post-procedure. For cases where direct evaluation was not possible after 24 h, skin reactions were assessed through an interview with the owner. For patients available for follow-up 1 week after the procedure, skin reactions were assessed by comparing photographs taken at the time of treatment with the current skin condition. If a follow-up visit was not possible, adverse reactions at the treatment site were assessed through a telephone interview 1 week after the procedure.

### 2.3 Pain assessment scoring method

#### 2.3.1 Video recording of the cryoanesthesia procedures and video-based pain assessment

**Figure 3:**
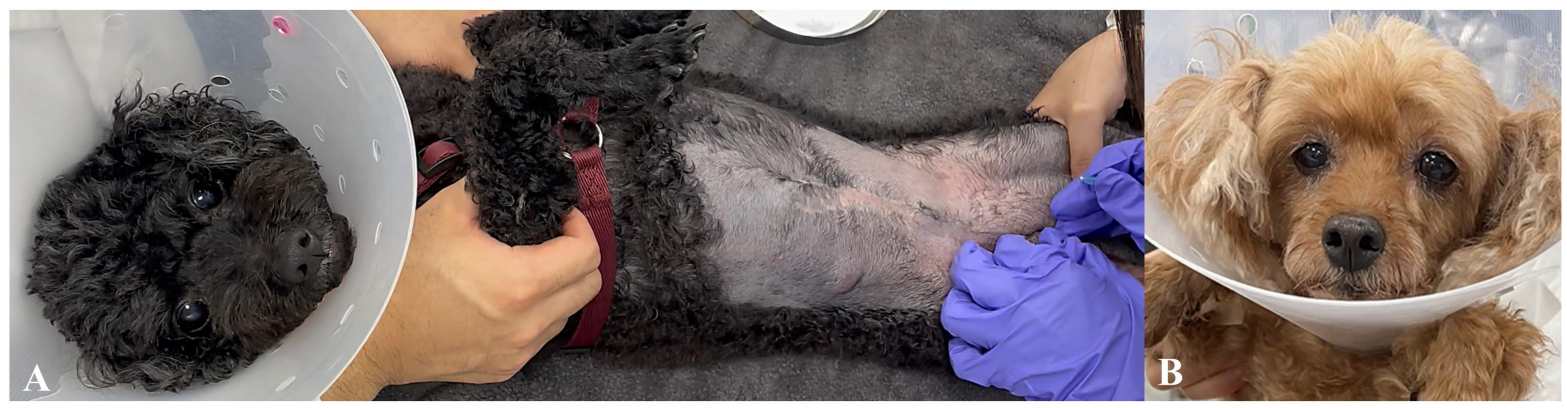
Video recording for visual analog scale (VAS) and Modified Pain Score (mPS) assessment during procedure **(A)** Entire body video capture to assess body movement during procedure for pain evaluation. **(B)** Close-up of facial expressions.

**Figure 4:**
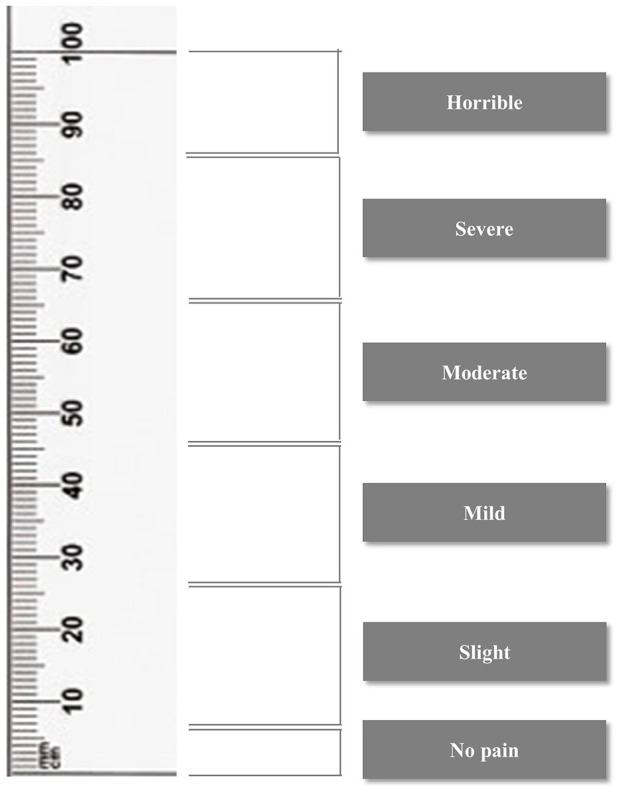
Visual analog scale. The visual analog scale allows pain to be assessed and graded according to the score.

All procedures were recorded using mobile phones. The recording started just before needle insertion and continued until the moment the needle was fully inserted. The video captured the entire body, including facial expressions, to observe full-body movement during the procedure (Figure 3, Supplementary Video 2). Through analysis of the recorded video footage, pain was assessed using two scoring systems: the modified Pain Score (mPS) (Table 1) and the Visual Analog Scale (VAS) (Figure 4).

**Table 1:** Modified pain scoring system for assessing the degree of pain in animals during needle insertion.

**Criteria**	Score		
	**No reaction**	**Mild to moderate**	**Severe**
Vocalization	0	1	2
Head turning toward the injection	0	1	2
General movement	0	1	2
Eye movement toward the injection	0	1	2
Aggression	0	1	2
	**Absent**		**Present**
Twitching	0		1
Panting	0		1

Adapted from Flecknell et al. (21), van Oostrom and Knowles (3), Crisi et al. (20), and Tomich et al. (19).

Pain assessments were independently performed by three veterinarians with more than two years of experience in emergency and critical care, all of whom were blinded to treatment allocation. Only the moment of needle insertion was evaluated. Prior to scoring, observers underwent standardized training, including review of the scoring criteria and calibration using sample videos to ensure consistent interpretation. Mean scores across the three independent observers were used for analysis to minimize individual observer bias and to obtain a more robust estimate of pain-related behavioral responses.

#### 2.3.2 Modified pain score

The modified pain score (mPS) was assessed based on adverse reactions observed immediately following needle insertion and was adapted from previously published procedure-specific behavioral scoring systems to capture immediate pain-related responses (3, 19–21). For each case, seven reactions were evaluated: vocalization, head movement toward the injection site, general movement, eye movement toward the injection site, aggression, twitching, and panting. Each reaction was scored as no reaction (0), mild to moderate (1), or severe (2), except for twitching and panting, which were assessed as either absent (0) or present (1). Accordingly, the total mPS ranged from 0 to 12 (Table 1). For responses toward the injection site (head turning and/or eye movement), mild to moderate reactions were defined as brief or partial head or eye movements toward the injection site without accompanying body withdrawal or resistance, whereas severe reactions were characterized by rapid and pronounced head turning or sustained eye movement with clear avoidance behavior. For vocalization, mild to moderate responses consisted of brief, low-intensity vocal sounds, whereas severe responses were defined as sustained, high-pitched, or repeated vocalizations. For the movement category, mild to moderate responses were defined as localized bodily responses or limited movement that did not substantially interfere with the procedure, while severe responses involved marked whole-body withdrawal or pronounced movement clearly disrupting the procedure. For aggression, mild to moderate responses were defined as defensive posturing or mild threat-related behaviors, whereas severe responses included biting attempts or overt aggressive movements.

Among these reactions, panting was defined as open-mouth breathing characterized by rapid and shallow respirations (respiratory rate ≥120 breaths/min). Because room temperature remained consistent before and after application of the vapocoolant spray, panting observed within 5 s after stimulation was unlikely to result from ambient temperature changes and was therefore recorded as an adverse reaction rather than a thermoregulatory response.

#### 2.3.3 Visual analog scale

The VAS was a horizontal line ranging from 0 mm to 100 mm, with the left end representing no pain and the right end indicating the worst possible pain (22). For each procedure, observers marked the horizontal line at the point corresponding to the dog's perceived pain response. To facilitate interpretation, VAS scores were categorized into the following pain grades: 0–4.9 mm (no pain), 5–24.9 mm (slight pain), 25–44.9 mm (mild pain), 45–64.9 mm (moderate pain), 65–84.9 mm (severe pain), and 85–100 mm (horrible pain) (Figure 4). This classification allows for a standardized evaluation of pain intensity based on the VAS score.

### 2.4 Statistical analysis

Statistical analyses were performed using a computer-based statistical program (Stata version 18; StataCorp LLC, College Station, TX, USA). Generalized estimating equations (GEE) were applied to account for within-subject correlation arising from repeated procedures in some dogs. Group effects were evaluated using model-based regression coefficients relative to the control group, and Wald χ^2^ statistics were used for hypothesis testing. All tests were two-tailed, and statistical significance was set at *p* < 0.05 (Tables 2, 3).

**Table 2:** Modified pain scores of dogs to needle insertion between the treatment group and the control group.

**Indication**	Mean			***p***-***value*** for generalized estimating equations		
	**Control**	**Group A**	**Group B**	**Control, Group A**	**Control, Group B**	**Group A, Group B**
FNA	2.6	2.1	1.5	0.324 (0.97)	0.068 (3.32)	0.008^*^ (6.89)
Jugular venipuncture	1	0.3	0.2	0.099 (2.71)	0.042^*^ (4.10)	0.675 (0.18)
Centesis	3.7	1.3	0.8	< 0.001^*^ (17.14)	< 0.001^*^ (36.46)	0.371 (0.80)

^*^p-value < 0.05 indicates significance. Models were estimated using generalized estimating equations with exchangeable (FNA, centesis) or independent (jugular venipuncture) working correlation structures, as appropriate. Values are presented as *p*-values, with corresponding Wald χ^2^ statistics shown in parentheses.

**Table 3:** VAS of dogs to needle insertion between the treatment group and the control group.

**Indication**	Mean			***p***-***value*** for generalized estimating equations		
	**Control**	**Group A**	**Group B**	**Control, Group A**	**Control, Group B**	**Group A, Group B**
FNA	27.6 mm Mild	22.6 mm Slight	12.3 mm Slight	0.419 (0.65)	0.010^*^ (7.31)	0.094 (2.79)
Jugular venipuncture	11.0 mm Slight	8.0 mm Slight	3.6 mm No pain	0.549 (0.36)	0.138 (2.19)	0.217 (1.52)
Centesis	28.0 mm Mild	10.3 mm Slight	5.7 mm Slight	0.050^*^ (6.49)	0.001^*^ (13.44)	0.032 (4.57)

^*^p-value < 0.05 indicates significance. Models were estimated using generalized estimating equations with exchangeable (FNA, centesis) or independent (jugular venipuncture) working correlation structures, as appropriate. Values are presented as *p*-values, with corresponding Wald χ^2^ statistics shown in parentheses.

## 3 Results

### 3.1 Case identification

A total of 90 procedures were performed in 56 dogs in this study. These included 30 centesis procedures performed in 20 dogs, 30 fine-needle aspiration (FNA) procedures performed in 14 dogs, and 30 jugular venipuncture procedures performed in 25 dogs. Three dogs underwent two different types of needle-related procedures, specifically centesis and FNA. However, no dog was subjected to a predefined sequential protocol involving different procedural categories. When multiple procedures were performed in the same dog, they were conducted at separate anatomical sites and at different time points. Some dogs underwent more than one procedure during the study period, and repeated measurements within the same individual were accounted for in the statistical analysis based on the immediate behavioral response observed at the moment of needle insertion. Detailed information on individual dogs, procedure types, anatomical sites, and pain scores is provided in Supplementary Table 1. Across indications, dogs undergoing centesis and FNA had mean ages of approximately 9.3–10.7 years and mean body weights ranging from 6.8 to 14.0 kg. In contrast, dogs undergoing jugular venipuncture were younger, with mean ages of approximately 5.0–5.5 years and mean body weights ranging from 32.6 to 36.4 kg. Sex distributions were comparable across procedure groups (Table 4). For centesis (cystocentesis, thoracentesis, abdominocentesis), no statistically significant differences were observed among the control, Group A, and Group B with respect to age (*p* = 0.748) and body weight (*p* = 0.117). Similarly, in FNA, age (*p* = 0.878) and body weight (*p* = 0.740) did not differ significantly among the groups. In jugular venipuncture, no statistically significant differences were found among the groups for age (*p* = 0.772) and body weight (*p* = 0.440; Table 4).

**Table 4:** Overall patient's signalment.

**Indication**	**Procedures**	**Age (years)**	**Body weight (kg)**	**Sex (IF/SF/IM/CM)**
Centesis	Control	10.4 (7–13)	10.5 (2.2–32)	1/3/0/6
	Group A (2 °C 2 s)	10.7 (5–14)	14 (5.2–32)	1/5/1/3
	Group B (2 °C 5 s)	9.8 (5–14)	6.8 (2.2–13)	3/2/0/5
*p-value*		0.748	0.117	–
FNA	Control	9.3 (3–16)	10.34 (3–38)	2/6/0/2
	Group A (2 °C 2 s)	10.5 (3–16)	10.25 (3.2–38)	2/6/0/2
	Group B (2 °C 5 s)	9.8 (3–16)	11.1 (4.3–38)	2/7/0/1
*p-value*		0.878	0.740	–
Jugular venipuncture	Control	5.3 (3–8)	36.4 (25–51)	1/3/1/5
	Group A (2 °C 2 s)	5.0 (3–8)	35.7 (25–54)	0/5/1/4
	Group B (2 °C 5 s)	5.5 (3–8)	32.6 (25.8–50)	0/7/0/3
*p-value*	–	0.772	0.440	–

BW, body weight; IF, intact female; SF, spayed female; IM, intact male; CM, castrated male; FNA, fine-needle aspiration. Age (years): Values represent mean (range), Body weight (kg): Values represent mean (range).

Dogs undergoing jugular venipuncture were healthy individuals enrolled through a blood donor program. In contrast, dogs included in the FNA and centesis groups were client-owned hospital cases presenting for diagnostic evaluation. All FNA and centesis procedures were performed in clinically stable patients and were planned as part of a diagnostic workup rather than conducted under emergency conditions.

### 3.2 Pain assessment results

Inter-observer agreement for VAS scoring was high among the three veterinarians (ICC = 0.94). The following sections describe procedure-specific pain outcomes based on both mPS and VAS assessments.

#### 3.2.1 Centesis procedures

Among the 30 centesis procedures, cystocentesis was the most frequently performed procedure (*n* = 21), followed by abdominocentesis (*n* = 6) and thoracentesis (*n* = 3). In the control group, mean pain scores and VAS values were descriptively higher for abdominal centesis (mean pain score, 5.0; mean VAS, 4.33) compared with cystocentesis (mean pain score, 3.17; mean VAS, 2.17), whereas thoracentesis was represented by a single case (mean pain score, 3.0; mean VAS, 2.0).

**Figure 5:**
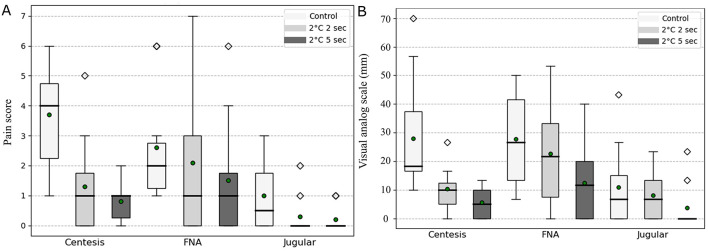
Box plot presentation of the Vapocoolant spray group and control group for the modified pain score (mPS) and the visual analog scale (VAS). In centesis, fine-needle aspiration (FNA), and jugular venipuncture, the mPS and VAS were assessed in the Vapocoolant spray application group and compared with those of the control group. This graph illustrates the reduction in pain levels in groups A and B compared with the control group. **(A)** This plot presents the mPS results. A statistically significant difference in mPS was observed only in centesis (*p* = 0.001). **(B)** This plot presents the VAS results. Similar to the mPS, a statistically significant difference was observed only in centesis (*p* = 0.001). This box plot represents the following statistical features: green circles indicate the mean values, the upper and lower whiskers represent the range of non-outlier data, the bold horizontal line within the box indicates the median, the box represents the interquartile range, and the diamond shapes denote outlier points.

The mean mPS out of a total of 12 points was 3.7 ± 1.7 in the control group, 1.3 ± 1.6 in group A, and 0.8 ± 0.6 in group B, respectively (Figure 5A). Using GEE, pairwise comparisons showed significantly lower mPS in group A compared with the control group (*p* = 0.001) and in group B compared with the control group (*p* = 0.001), whereas no significant difference was detected between group A and group B (*p* = 0.371; Table 2).

In the VAS analysis, the control group had a mean score of 28.0 ± 21.0 mm, indicating mild pain. When cryoanesthesia was applied at 2 °C for 2 s, the mean VAS score was 10.3 ± 7.6 mm, indicating slight pain. Cryoanesthesia applied at 2 °C for 5 s resulted in an even lower mean score of 5.7 ± 5.7 mm, also indicating slight pain (Figure 5B). GEE-based pairwise comparisons demonstrated lower VAS scores in group A compared with the control group (*p* = 0.050) and in group B compared with the control group (*p* = 0.001). A significant difference was also observed between group A and group B (*p* = 0.032; Table 3).

#### 3.2.2 FNA procedures

A total of 30 FNA procedures were performed in 14 dogs. Because some dogs underwent multiple FNA procedures at different anatomical sites, FNA locations were summarized on a per-procedure basis and included the mammary gland, lymph nodes (mediastinal or popliteal), skin or subcutaneous masses, and other soft tissue or limb-associated lesions. The number of FNA procedures performed per dog ranged from 1 to 5.

The mean mPS was 2.6 ± 1.8 in the control group, 2.1 ± 2.6 in group A, and 1.5 ± 2.0 in group B (Figure 5A). Using GEE, pairwise comparisons showed no significant difference between the control group and group A (*p* = 0.324) or between the control group and group B (*p* = 0.068). In contrast, mPS was significantly lower in group B than in group A (*p* = 0.008; Table 2).

The mean VAS scores were 27.6 ± 15.5 mm in the control group, 22.6 ± 18.8 mm in group A, and 12.3 ± 13.3 mm in group B (Figure 5B). GEE-based pairwise comparisons showed no significant difference between the control group and group A (*p* = 0.419), whereas VAS scores were significantly lower in group B compared with the control group (*p* = 0.010). No significant difference was observed between groups A and B (*p* = 0.094; Table 3).

#### 3.2.3 Jugular venipuncture procedures

The mean mPS was 1.0 ± 1.2 in the control group, 0.3 ± 0.6 in group A, and 0.2 ± 0.4 in group B (Figure 5A). Using GEE, pairwise comparisons showed no significant difference between the control group and group A (*p* = 0.099) or between the control group and group B (*p* = 0.042). No significant difference was observed between groups A and B (*p* = 0.675; Table 2).

The mean VAS score was 11.0 ± 14.4 mm in the control group, 8.0 ± 8.5 mm in group A, and 3.6 ± 8.1 mm in group B (Figure 5B). GEE-based pairwise comparisons showed no significant difference between the control group and group A (*p* = 0.549) or between the control group and group B (*p* = 0.138). No significant difference was observed between groups A and B (*p* = 0.217; Table 3).

### 3.3 Complications of vapocoolant spray

Adverse event assessments were performed in all cases. For centesis and FNA procedures conducted in hospitalized patients, immediate and 24-h assessments were performed by veterinarians via direct visual inspection of the procedure sites. For jugular venipuncture procedures performed in blood donor dogs, the 24-h assessment was conducted through owner interviews. In all cases, the 1-week adverse event assessment was performed by a veterinarian based on owner interviews and photographic documentation. No adverse skin reactions or complications were observed at any assessment time point, including immediately after the procedure, 24 h post-procedure, and 1 week post-procedure.

## 4 Discussion

In this study, we evaluated the analgesic effects of a novel vapocoolant device (VetEase^®^, Recensemedical, Hwaseong-si, Republic of Korea) during needle-related procedures in companion animals. The device was developed to rapidly reduce skin temperature by utilizing cooling generated from the rapid expansion of compressed CO_2_ gas as it passes through the nozzle.

Traditional vapocoolant sprays, including ethyl-chloride and fluoromethane-based formulations, have been widely used in both human and veterinary medicine (8, 10–14, 23). Although these agents induce immediate cooling through rapid evaporation, their simple spray-based delivery results in substantial operator-dependent variability, with cooling intensity and duration potentially influenced by factors such as spraying distance, angle, and application time (14).

Unlike liquid-based sprays, CO_2_ gas does not remain on the skin surface after application. Accordingly, the cooling effect appears to be transient and decreases rapidly after application. Nevertheless, a strong and localized cooling effect was observed on the skin surface at the moment of CO_2_ gas release. The novel CO_2_ vapocoolant device is designed to standardize spraying distance, angle, target temperature, and application duration, minimizing operator-related variability. Moreover, while formal validation studies using tissue temperature probes have not yet been reported, the device incorporates a skin surface temperature sensor and is designed to maintain the measured skin surface temperature within a controlled range of −5 °C to 5 °C throughout application, thereby providing more predictable and reproducible cooling. This controlled and localized cooling mechanism may help reduce adverse effects such as swelling, erythema, pain, discoloration, and tissue necrosis, offering a more targeted and reliable approach to procedural pain management compared with conventional vapocoolant sprays.

Currently, there is no established consensus on a standardized protocol for achieving local anesthesia with a vapocoolant spray in dogs, including optimal temperature, application time, and related parameters. Moreover, this study represents the first application of this novel CO_2_ vapocoolant device in companion dogs. To determine the application temperature, the approach was extrapolated from previous human literature, in which the same CO_2_-based vapocoolant device used in the present study was applied at 2 °C during tattoo removal (15). In contrast, the application time was guided by previous studies employing ethyl chloride–based vapocoolant sprays, which reported direct application to the skin surface for 5 s in children and up to 15 s in horses (11, 23, 24). Although these studies utilized a different vapocoolant system, they were used to provide contextual guidance on tolerable and safe exposure durations for direct skin application across species, rather than to imply equivalence between vapocoolant systems. Furthermore, considering that dogs generally have a thinner epidermal layer compared to humans (25) and that the temperature range capable of inducing frostbite in canine skin has been reported to range from −10.6 °C to 2.2 °C (26), the final application temperature was set at 2 °C. For the application time, a 5-s duration, consistent with that used in pediatric studies, was initially selected. However, since some dogs were sensitive to the noise and pressure generated during spraying and exhibited agitation or anxiety during the 5-s application, an additional condition of a 2-s application, which is a shorter duration than 5 s, was included. The analgesic effects of the 2-s and 5-s application durations were then compared.

During centesis procedures, both Group A and Group B demonstrated lower pain scores than the control group, with statistically significant reductions observed in both mPS and VAS. No statistically significant difference was observed between the two cryoanesthesia application conditions. These findings indicate that the application of cryoanesthesia itself is effective in reducing procedure-related pain during centesis. Notably, although Group B tended to show lower pain scores than Group A during centesis, no statistically significant difference was detected between the two application durations for mPS. This finding may reflect a ceiling effect of the pain scoring system, whereby pain responses had already been substantially attenuated, limiting the ability to detect additional differences between treatment conditions. In other words, once pain scores approach the lower end of the scale, the sensitivity of mPS to distinguish further incremental reductions may be limited.

Several factors were considered to explain the lack of significant analgesic effects observed during FNA and jugular venipuncture. The first factor was the depth of the cryoanesthetic effect provided by the novel vapocoolant spray used in this study. At the time of the study, there were no published data describing the extent to which this vapocoolant spray delivered cryoanesthetic effects in either human or canine skin. In the case of FNA, the initial pain during needle insertion was similar to that observed during centesis. However, unlike centesis, in which the needle passes through the skin and muscle layers and is positioned within a cavity such as the bladder, thoracic cavity, or abdominal cavity, the entire needle is positioned within the tumor tissue during FNA. As a result, the needle continuously stimulates tissues and nerve fibers within the tumor, which could potentially intensify pain compared with centesis. Furthermore, direct needle insertion through tumor lesions may exacerbate local inflammatory responses, increasing pain sensitivity in the affected areas and potentially resulting in greater pain compared with non-lesion sites (27–29). In addition, although pain scores differed significantly between the 2-s and 5-s application groups in FNA, the comparison between the 5-s application and the control group did not reach statistical significance. This finding indicates that while increasing the application duration may enhance the magnitude of pain attenuation, surface-applied cryoanesthesia alone may be insufficient to fully suppress pain generated within deeper tissue. Consequently, there may have been limitations in alleviating pain induced within the tissue, which may explain the relatively reduced analgesic effect observed during FNA.

Secondly, in the case of jugular venipuncture, it should be noted that dogs weighing ≥25 kg were intentionally enrolled in this study. This decision reflects the clinical environment of our institution, where a blood donation center is operated and jugular venipuncture using relatively large-gauge needles in large-breed dogs is frequently performed. In such settings, larger-gauge needles than the commonly used 23G are often required to ensure adequate blood flow during blood collection. Given concerns that increased needle diameter may exacerbate tissue stimulation and procedural discomfort, this study was designed to evaluate the analgesic effects of the vapocoolant device under clinically relevant conditions in which stronger pain responses could be anticipated. In the present study, despite the use of a relatively larger gauge needle compared with centesis or FNA, the initial pain response during needle insertion in the control group was less pronounced than that observed during centesis or FNA. A significant reduction in pain scores was observed in Group B compared with the control group; however, no statistically significant difference was detected in VAS assessments. Although there is no objective evidence to suggest that breed or body size directly reduces pain sensitivity, the lack of statistical significance observed in VAS measurements may, in part, reflect the subjective nature of this assessment tool. Previous studies have demonstrated that veterinarians' perceptions of pain sensitivity in dogs, including perceptions related to larger breeds, do not consistently align with objectively measured pain sensitivity (30); therefore, such perception-related bias may have influenced VAS scoring in the present study. Accordingly, differences observed between pain scoring systems may reflect the inherent characteristics of each assessment method rather than true differences in analgesic efficacy. Additionally, the relatively thicker skin at the neck may have limited the depth of cooling achieved, potentially resulting in a less effective cryoanesthetic effect during jugular venipuncture (25, 31).

This novel vapocoolant spray is relatively simple to apply and safe. However, certain important considerations should be taken into account. Firstly, prolonged application has been associated with adverse effects such as allergic contact dermatitis, hypopigmentation, and atrophic scarring, particularly in individuals with impaired circulation who are at higher risk (6). In this study, no skin complications were observed in any of the dogs subjected to 2 °C application for either 2 or 5 s, as assessed both immediately after the procedure and during the 1-week follow-up. Secondly, novel vapocoolant spray produces a relatively loud noise during operation, which can cause startle or flinching reactions in patients. While this is not a major concern in humans, some dogs, particularly those that are noise-sensitive, exhibited agitation or anxiety during the procedure. To address this issue, 2 s of application time was also attempted as a reduction from the initial 5-s protocol. Nevertheless, except for centesis, the 2-s application generally did not demonstrate significant analgesic effects across the evaluated procedures.

This study has several limitations. First, pain evaluation in animals is inherently challenging, as animals exhibit specific responses but cannot self-report pain, requiring interpretation by observers. Dogs typically respond to noxious stimuli by turning their head or eyes, panting, moving, or vocalizing (32, 33). In this study, two pain response scoring systems, mPS and VAS, were selected to assess pain. The mPS was adapted based on pain-scoring frameworks previously described in the literature and was designed to allow a more objective assessment of specific behavioral responses using a quantitative scale (3, 19–21). The VAS is a widely recognized tool for assessing pain intensity and is known for its reliability and sensitivity in evaluating pain in human subjects (22, 34). To ensure objectivity and minimize potential bias, three veterinarians independently evaluated the VAS, and the average of their evaluations was used. Although dogs that exhibited pronounced hypersensitivity to spray-related noise or pressure were excluded from the study according to the predefined exclusion criteria, limitations remained in fully distinguishing non-pain-related sensory responses, such as reactions to spray noise or pressure, from pain-related behaviors. Consequently, the potential influence of such responses on the observed procedural reactions cannot be excluded. In addition, objective physiological pain markers were not included in the present study. Measures such as heart rate variability, salivary cortisol concentration, pupil dilation, and thermal imaging could provide complementary information regarding analgesic efficacy. However, in a real-world clinical setting, the simultaneous acquisition of such physiological measurements during pain assessment is often impractical, as additional testing may disrupt routine clinical workflows or impose unnecessary stress on conscious patients. Notably, the vapocoolant spray evaluated in this study produces a rapid-onset analgesic effect that occurs within seconds after application and dissipates shortly thereafter; therefore, even brief delays in physiological data acquisition may limit the ability to accurately capture procedure-related pain responses. For these reasons, physiological measurements requiring specialized equipment, extended handling, or additional restraint were not incorporated into the study design. Nevertheless, the inclusion of objective physiological pain markers would strengthen the evidence base, and future studies employing multimodal pain assessment approaches with high temporal resolution are warranted.

Second, procedures were performed by multiple veterinarians, and individual operator skill levels were neither standardized nor formally assessed. Although this may have contributed to variability in pain perception, all operators followed standardized institutional protocols for needle-based procedures. In addition, treatment group allocation was independent of operator assignment, which may have mitigated systematic bias related to procedural technique. However, because operators performing the spray application and needle insertion were not blinded to treatment allocation, potential expectation bias or subtle differences in handling cannot be completely excluded. Accordingly, operator-related variability and the single-blind study design represent limitations that should be considered when interpreting the results.

Lastly, for each indication and temperature condition, only 10 cases were included in the study. This limited sample size may not have been sufficient to detect significant differences in responses, and future studies with larger patient cohorts are therefore needed to obtain more robust and conclusive findings. In addition, although no adverse skin reactions or complications were observed, safety assessments were limited to short-term follow-up and were partly based on owner-reported outcomes, which may limit definitive conclusions regarding long-term safety.

The present study focused on evaluating the clinical applicability of the newly developed cryotherapy device by assessing its analgesic effects at a predefined target temperature with different exposure durations. Because the main aim of this study was to evaluate the clinical applicability and feasibility of the device in a practical setting, direct comparisons with existing vapocoolant sprays, which differ in composition, cooling mechanisms, and recommended application times, were not included in the study design. Nevertheless, such comparisons are important for determining the device's relative clinical value, and future research should include direct comparative evaluations with conventional vapocoolant sprays as well as other topical anesthetics to more clearly define its relative analgesic efficacy and safety profile.

## 5 Conclusion

This study explored the use of a novel CO_2_ vapocoolant spray to alleviate pain associated with selected needle-related procedures through cryoanesthesia. A consistent and statistically significant reduction in pain responses was observed during centesis, with the 2 °C for 5 s protocol showing the most favorable performance under the conditions evaluated. For FNA and jugular venipuncture, reductions in pain measures were observed in some comparisons; however, these findings were not consistent across outcomes and comparisons, and therefore provide limited evidence to support definitive clinical conclusions. Overall, the present results suggest that this vapocoolant spray may have potential clinical applicability, particularly for centesis-related procedures, while larger controlled trials are warranted to clarify its effectiveness across other indications and to define its role relative to existing analgesic strategies.

## Data Availability

The raw data supporting the results of this article are included in the article and/or the Supplementary material.

## Supplementary material

Supplementary Table 1 Detailed information on individual dogs, including procedure type, anatomical site, and corresponding pain scores.

Supplementary Video 1 Demonstration of the device setup procedure, showing how to set the spray temperature and application duration to 2°C for 5 seconds.

Supplementary Video 2 Representative video recording used for video-based pain assessment during needle insertion.
