# Supplementary material for: The efficacy of a novel CO2 topical vapocoolant spray for reducing needle-related pain in dogs
Source: Front Vet Sci. 2026 Mar 10;13:1754998. doi: 10.3389/fvets.2026.1754998 (PMC13011815; doi:10.3389/fvets.2026.1754998)
Supplement: Supplementary Table 1 — Detailed information on individual dogs, including procedure type, anatomical site, and corresponding pain scores. [file Table_1.docx]

`

| **Dog** | **Indication** | **site of application** | **needle gauge** | **type** | **pain_score** | **Vet 1 (VAS)** | **Vet 2(VAS)** | **Vet 3 (VAS)** | **SF-GCPS (0~24)** |
| --- | --- | --- | --- | --- | --- | --- | --- | --- | --- |
| Maru-0 | Jugular venipuncture | Lt. jugular vein | 16G | 2℃ 2sec | 1 | 1 | 2 | 1 | 0 |
| Kkotbun | Jugular venipuncture | Rt. Jugular vein | 16G | 2℃ 2sec | 0 | 0 | 0 | 0 | 0 |
| Soja | Jugular venipuncture | Lt. jugular vein | 16G | 2℃ 2sec | 0 | 0 | 1 | 0 | 0 |
| Ken | Jugular venipuncture | Lt. jugular vein | 16G | Control | 1 | 0 | 0 | 0 | 0 |
| Ken | Jugular venipuncture | Lt. jugular vein | 16G | 2℃ 2sec | 0 | 0 | 0 | 0 | 0 |
| Calix | Jugular venipuncture | Lt. jugular vein | 16G | Control | 1 | 1 | 0 | 0 | 0 |
| Biang | Jugular venipuncture | Lt. jugular vein | 16G | 2℃ 5sec | 0 | 0 | 0 | 0 | 0 |
| Mare | Jugular venipuncture | Lt. jugular vein | 16G | 2℃ 5sec | 0 | 0 | 0 | 0 | 0 |
| Haru | Jugular venipuncture | Lt. jugular vein | 16G | 2℃ 5sec | 0 | 0 | 0 | 0 | 0 |
| Bori | Jugular venipuncture | Lt. jugular vein | 16G | Control | 3 | 4 | 5 | 4 | 0 |
| Bori | Jugular venipuncture | Lt. jugular vein | 16G | 2℃ 5sec | 0 | 0 | 0 | 0 | 0 |
| Kkotnim | Jugular venipuncture | Lt. jugular vein | 16G | 2℃ 5sec | 1 | 2 | 3 | 2 | 0 |
| Tommy | Jugular venipuncture | Rt. Jugular vein | 16G | 2℃ 5sec | 0 | 0 | 0 | 0 | 0 |
| Sunny | Jugular venipuncture | Lt. jugular vein | 16G | 2℃ 5sec | 0 | 0 | 0 | 0 | 0 |
| Lulu | Jugular venipuncture | Lt. jugular vein | 16G | 2℃ 5sec | 0 | 0 | 0 | 0 | 0 |
| Kungi | Jugular venipuncture | Lt. jugular vein | 16G | 2℃ 5sec | 1 | 1 | 2 | 1 | 0 |
| Manju | Jugular venipuncture | Lt. jugular vein | 16G | 2℃ 5sec | 0 | 0 | 0 | 0 | 0 |
| Haengun | Jugular venipuncture | Rt. jugular vein | 16G | 2℃ 2sec | 0 | 1 | 2 | 1 | 0 |
| Huimang | Jugular venipuncture | Lt. jugular vein | 16G | 2℃ 2sec | 2 | 2 | 3 | 2 | 0 |
| Huimang | Jugular venipuncture | Lt. jugular vein | 16G | Control | 3 | 2 | 2 | 1 | 0 |
| Onbi | Jugular venipuncture | Lt. jugular vein | 16G | 2℃ 2sec | 0 | 0 | 0 | 0 | 0 |
| Onbi | Jugular venipuncture | Lt. jugular vein | 16G | Control | 0 | 0 | 0 | 0 | 0 |
| Gold | Jugular venipuncture | Rt. jugular vein | 16G | Control | 2 | 3 | 3 | 2 | 0 |
| Rich | Jugular venipuncture | Lt. jugular vein | 16G | Control | 0 | 0 | 0 | 0 | 0 |
| Ena | Jugular venipuncture | Lt. jugular vein | 16G | 2℃ 2sec | 0 | 0 | 0 | 0 | 0 |
| Oreo | Jugular venipuncture | Lt. jugular vein | 16G | Control | 0 | 1 | 1 | 1 | 0 |
| Taylor | Jugular venipuncture | Lt. jugular vein | 16G | 2℃ 2sec | 0 | 1 | 1 | 1 | 0 |
| Taylor | Jugular venipuncture | Lt. jugular vein | 16G | Control | 0 | 1 | 2 | 0 | 0 |
| Lucky | Jugular venipuncture | Lt. jugular vein | 16G | Control | 0 | 0 | 0 | 0 | 0 |
| Jerry | Jugular venipuncture | Rt. Jugular vein | 16G | 2℃ 2sec | 0 | 1 | 2 | 2 | 0 |
| Baekdusan | Centesis | Cystocentesis | 23G | Control | 4 | 5 | 6 | 6 | 1 |
| Choco | Centesis | Abdominocentesis | 23G | Control | 6 | 6 | 8 | 7 | 5 |
| Choco | Centesis | Abdominocentesis | 23G | 2℃ 5sec | 2 | 1 | 2 | 1 | 5 |
| Suji | Centesis | Cystocentesis | 23G | 2℃ 2sec | 2 | 1 | 1 | 1 | 1 |
| Suji | Centesis | Cystocentesis | 23G | Control | 4 | 2 | 2 | 1 | 1 |
| Yanbyeol | Centesis | Thrococentesis | 23G | 2℃ 2sec | 0 | 1 | 1 | 1 | 2 |
| Ddungsun | Centesis | Cystocentesis | 23G | 2℃ 2sec | 0 | 1 | 2 | 0 | 1 |
| Ddungsun | Centesis | Cystocentesis | 23G | 2℃ 5sec | 0 | 0 | 0 | 0 | 1 |
| Sikssiki | Centesis | Cystocentesis | 23G | 2℃ 5sec | 0 | 0 | 0 | 0 | 1 |
| Jelly | Centesis | Cystocentesis | 23G | 2℃ 5sec | 1 | 1 | 1 | 0 | 3 |
| Gomi | Centesis | Cystocentesis | 23G | 2℃ 5sec | 1 | 0 | 0 | 0 | 3 |
| Aebong | Centesis | Cystocentesis | 23G | Control | 2 | 1 | 2 | 2 | 3 |
| Aebong | Centesis | Cystocentesis | 23G | 2℃ 5sec | 1 | 1 | 1 | 1 | 3 |
| Maru-1 | Centesis | Abdominocentesis | 23G | 2℃ 5sec | 1 | 1 | 2 | 1 | 5 |
| Maru-1 | Centesis | Cystocentesis | 23G | Control | 2 | 2 | 2 | 2 | 5 |
| Maru-1 | Centesis | Abdominocentesis | 23G | Control | 4 | 1 | 3 | 1 | 5 |
| Shinji | Centesis | Thrococentesis | 23G | Control | 3 | 2 | 2 | 2 | 1 |
| Shinji | Centesis | Thrococentesis | 23G | 2℃ 5sec | 0 | 0 | 0 | 0 | 1 |
| Junpi | Centesis | Abdominocentesis | 23G | 2℃ 2sec | 0 | 0 | 1 | 0 | 1 |
| Junpi | Centesis | Abdominocentesis | 23G | Control | 5 | 4 | 5 | 4 | 1 |
| Maru-2 | Centesis | Cystocentesis | 23G | Control | 1 | 1 | 1 | 1 | 0 |
| Maru-2 | Centesis | Cystocentesis | 23G | 2℃ 2sec | 1 | 1 | 2 | 2 | 0 |
| Gwiyom | Centesis | Cystocentesis | 23G | 2℃ 2sec | 5 | 2 | 4 | 2 | 5 |
| Gwiyom | Centesis | Cystocentesis | 23G | Control | 6 | 1 | 1 | 1 | 5 |
| Baekeok | Centesis | Cystocentesis | 23G | 2℃ 2sec | 3 | 1 | 1 | 1 | 3 |
| Cookie | Centesis | Cystocentesis | 23G | 2℃ 2sec | 1 | 1 | 2 | 1 | 3 |
| Sundol | Centesis | Cystocentesis | 23G | 2℃ 5sec | 1 | 1 | 1 | 1 | 3 |
| Garu | Centesis | Cystocentesis | 23G | 2℃ 2sec | 1 | 0 | 1 | 0 | 5 |
| Alice | Centesis | Cystocentesis | 23G | 2℃ 5sec | 1 | 0 | 1 | 0 | 0 |
| Namu | Centesis | Cystocentesis | 23G | 2℃ 2sec | 0 | 0 | 0 | 0 | 3 |
| Bidong | FNA | mammary gland tumor | 23G | 2℃ 2sec | 1 | 3 | 4 | 3 | 5 |
| Bidong | FNA | mammary gland tumor | 23G | Control | 6 | 5 | 5 | 5 | 5 |
| Dubu | FNA | mammary gland tumor | 23G | 2℃ 2sec | 0 | 0 | 0 | 0 | 2 |
| Kkomi | FNA | popliteal lymph node | 23G | 2℃ 2sec | 1 | 2 | 3 | 2 | 2 |
| Kkomi | FNA | popliteal lymph node | 23G | Control | 1 | 4 | 4 | 5 | 0 |
| Gimmari | FNA | mediastinum lymph node | 23G | Control | 6 | 4 | 4 | 3 | 0 |
| Gimmari | FNA | mediastinum lymph node | 23G | 2℃ 2sec | 6 | 4 | 5 | 5 | 0 |
| Gimmari | FNA | mediastinum lymph node | 23G | 2℃ 5sec | 6 | 2 | 2 | 2 | 0 |
| Hosu | FNA | Rt. Metatarsal mass | 23G | 2℃ 2sec | 7 | 5 | 6 | 5 | 0 |
| Hosu | FNA | Rt. Metatarsal mass | 23G | 2℃ 5sec | 4 | 0 | 0 | 0 | 0 |
| Marong | FNA | abdomianl skin mass | 23G | 2℃ 5sec | 0 | 4 | 4 | 4 | 1 |
| Marong | FNA | abdomianl skin mass | 23G | Control | 2 | 4 | 4 | 5 | 1 |
| Marong | FNA | abdomianl skin mass | 23G | 2℃ 2sec | 0 | 0 | 1 | 1 | 1 |
| Gurumi | FNA | skin mass (Inguinal) | 23G | Control | 1 | 2 | 2 | 3 | 1 |
| Gurumi | FNA | hind limb skin mass | 23G | 2℃ 5sec | 1 | 2 | 2 | 2 | 1 |
| Gurumi | FNA | hind limb skin mass | 23G | 2℃ 2sec | 0 | 1 | 1 | 1 | 1 |
| Gurumi | FNA | hind limb skin mass | 23G | Control | 1 | 3 | 3 | 3 | 1 |
| Gurumi | FNA | skin mass (Inguinal) | 23G | 2℃ 5sec | 0 | 0 | 0 | 0 | 1 |
| Baro | FNA | mediastinum mass | 23G | Control | 3 | 2 | 3 | 2 | 1 |
| Baro | FNA | axillary lymph node | 23G | Control | 2 | 0 | 1 | 1 | 1 |
| Baro | FNA | mediastinum mass | 23G | 2℃ 2sec | 3 | 3 | 4 | 3 | 1 |
| Baro | FNA | mediastinum mass | 23G | 2℃ 5sec | 2 | 0 | 0 | 0 | 1 |
| Boksil | FNA | Mammary gland tumor | 23G | 2℃ 5sec | 1 | 2 | 2 | 2 | 2 |
| Cookie | FNA | Mammary gland tumor | 23G | Control | 2 | 1 | 1 | 1 | 3 |
| Cookie | FNA | Mammary gland tumor | 23G | 2℃ 5sec | 1 | 0 | 1 | 1 | 3 |
| Cookie | FNA | Mammary gland tumor | 23G | 2℃ 2sec | 3 | 1 | 3 | 2 | 3 |
| Maru-1 | FNA | forelimb mass | 23G | Control | 2 | 1 | 1 | 1 | 0 |
| Maru1 | FNA | forelimb mass | 23G | 2℃ 5sec | 0 | 1 | 2 | 2 | 0 |
| Maru1 | FNA | forelimb mass | 23G | 2℃ 2sec | 0 | 0 | 0 | 0 | 0 |
| Alice | FNA | Cervical skin mass | 23G | 2℃ 5sec | 0 | 0 | 0 | 0 | 0 |
